# Protein Hydrolysates from Fishery Processing By-Products: Production, Characteristics, Food Applications, and Challenges

**DOI:** 10.3390/foods12244470

**Published:** 2023-12-13

**Authors:** Mehdi Nikoo, Joe M. Regenstein, Mehran Yasemi

**Affiliations:** 1 Department of Pathobiology and Quality Control, Artemia and Aquaculture Research Institute, Urmia University, Urmia 57179-44514, Iran; 2 Department of Food Science, Cornell University, Ithaca, NY 14853-7201, USA; jmr9@cornell.edu; 3 Department of Fisheries, Institute of Agricultural Education and Extension, Agricultural Research, Education, and Extension Organization (AREEO), Tehran 19858-13111, Iran; yasemi_m@yahoo.com

**Keywords:** marine protein hydrolysates, hydrolysis variables, structure-function relations, antioxidant activity

## Abstract

Fish processing by-products such as frames, trimmings, and viscera of commercial fish species are rich in proteins. Thus, they could potentially be an economical source of proteins that may be used to obtain bioactive peptides and functional protein hydrolysates for the food and nutraceutical industries. The structure, composition, and biological activities of peptides and hydrolysates depend on the freshness and the actual composition of the material. Peptides isolated from fishery by-products showed antioxidant activity. Changes in hydrolysis parameters changed the sequence and properties of the peptides and determined their physiological functions. The optimization of the value of such peptides and the production costs must be considered for each particular source of marine by-products and for their specific food applications. This review will discuss the functional properties of fishery by-products prepared using hydrolysis and their potential food applications. It also reviews the structure–activity relationships of the antioxidant activity of peptides as well as challenges to the use of fishery by-products for protein hydrolysate production.

## 1. Introduction

In response to the recognition of the limits of marine sources and the crisis of food security, the production of aquatic food products is increasing and reached 179 million tonnes in 2018, of which 22 million tonnes (or 12%) were not used for human consumption [1]. To achieve the maximum use of by-products from fish processing industries (e.g., heads, frames, skin, trimmings, and viscera from fish; cephalothorax and shells from shrimp; heads and tentacles from squids; and shells and byssus threads from oysters and mussels), which account for 40–60% of the total weight, it is necessary to retain them in the food chain in line with a sustainable circular economy through the production of high-value biomolecules [2,3,4,5]. Of the different types of by-products that are produced after filleting, canning, packaging, etc., the heads, frames, and trimmings constitute >75% of the by-products’ weight and contain significant amounts of muscle residue that can be used for direct human consumption or can be converted to functional food ingredients. Despite this necessity, within the seafood sector, the increased use by-products is happening relatively slowly because the seafood industry focuses on its primary raw materials and products that require minimal processing [6]. 

One focus has been peptides from marine sources (fish, shellfish, and invertebrates) with antioxidant activity [7,8,9,10]. Antioxidant peptides have an essential role in inhibiting oxidation and scavenging free radicals. In the body, they may help fight aging and reduce food oxidation. These peptides have been prepared using enzymatic or autolytic hydrolysis or microbial fermentation [2]. Most peptides studied contain 2–10 amino acids (AAs), although some contain up to 20 AAs and had a molecular weight (MW) of 0.2 to 2 kDa [11,12,13,14]. Several proteases with different specificities for peptide bond cleavage and different hydrolysis conditions (temperature, time, pH, enzyme-to-substrate ratio, water/by-product ratio, and stirring rate) have been used to produce these marine by-product peptides [15,16,17,18]. Changes in hydrolysis conditions changed their antioxidant activities [19,20,21]. The antioxidant activity of peptides in foods has been associated with the scavenging of the free radicals formed during lipid peroxidation and metal chelation [22], which are dependent on the AA composition and sequence, size, hydrophobicity, and N- or C-terminal residues [23,24,25]. 

Marine protein hydrolysates derived using enzymatic hydrolysis showed antioxidant activity against free radicals and pro-oxidative metal ions. Thus, they may potentially be used as alternative antioxidants in foods and the human body to fight against free radical-mediated aging [26,27]. The structure of antioxidant peptides and protein hydrolysates from marine sources are highly variable depending on the types of by-products used as the initial protein source and the various operating parameters that affect the hydrolysis and the functional outcome. These by-products could be stabilized with non-oxidized products to preserve their freshness, in order to ensure lower deteriorative reactions during enzymatic hydrolysis, which is needed to obtain protein hydrolysates with acceptable organoleptic properties and storage stability. Marine protein hydrolysates and peptides have been shown to reduce the oxidation of both the lipids and proteins of seafood during storage, thus indicating antioxidant and anti-freezing effects [21,28,29,30]. 

Despite the suggested applications for protein hydrolysates from by-products in the food industry, several challenges have caused such products to be unable to be manufactured on a commercial scale. This review will discuss the production and characteristics of protein hydrolysates from fishery by-products derived using hydrolysis, the factors involved, and their potential applications to control/reduce the oxidative deterioration of seafood during storage. It will also review the structure–activity relationships of the antioxidant activity of peptides and the challenges of the industrial processing of by-products and the commercialization of protein hydrolysate production. 

## 2. Fish Protein Hydrolysate: Production and Processing Factors

The fish protein hydrolysates markets are anticipated to reach USD 558 million by 2025 with a compound annual growth rate (CAGR) of over 5% due to increasing demand for protein-based supplements, food formulations, infant nutrition, fertilizers, and aquafeeds due to their higher absorption or digestion [2]. These hydrolysates contain peptides with 2–10 amino acids or up to 20 amino acids and a molecular weight of <3 kDa, especially between 0.2 to 2 kDa [2,14]. The by-products of several commercial species such as tuna [17,20,31]; tilapia [12,32,33,34,35]; marine bony species [19,36,37]; small pelagics [38,39,40]; salmonids [15,16,41,42]; shrimp [18,43,44]; marine invertebrates such as mollusks [45,46,47], squid, and cuttlefish [48,49,50,51,52,53]; and underutilized fish [54,55] have been used as the sources for producing protein hydrolysates. By-products from these species showed differences in their compositions (i.e., different amounts of lipids, blood, proteins, undigested feed in their stomach or intestines, etc.), emphasizing the need to adjust the hydrolysis conditions for each source according to the inherent characteristics of the by-products (such as sorting by-products to obtain different fractions). Depending on the farming conditions or ocean water quality, there are some safety issues with by-products from different categories of aquatic species that should be considered. For example, undesirable metabolites and contaminants may enter the liquid phase in which peptides are formed, affecting the safety of the protein hydrolysates. Table 1 shows the yield, composition, safety concerns, and preventive measures for by-products from the main groups of aquatic species (fish, crustaceans, and invertebrates). 

**Figure 1:**
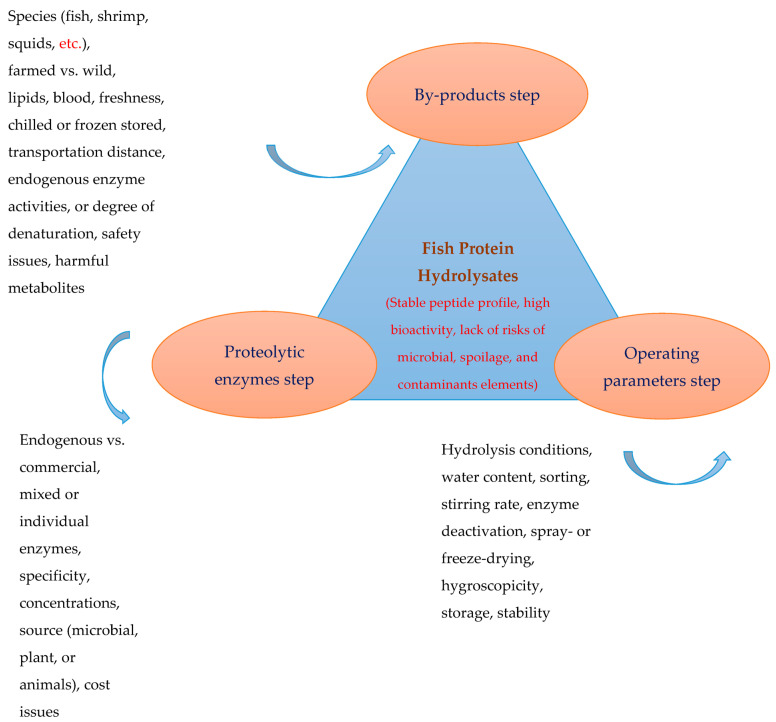
Main factors contributing to the enzymatic hydrolysis of fishery by-products and properties of the end products.

Enzymatic and autolytic hydrolysis and microbial fermentation have been used for converting the proteins of by-products into peptides with varying sizes and bioactivities [2,7,8,77]. The process of hydrolysis with commercial proteases can be controlled by selecting appropriate enzymes and adjusting the hydrolysis conditions (time, temperature, enzyme concentrations, water ratio, etc.). The final hydrolysates should be stable in terms of structure (such as peptide profile) and functions. Autolytic hydrolysis mediated by endogenous enzymes such as acid/aspartyl proteases (such as pepsin), serine proteases (trypsin and chymotrypsin), thiol/cysteine protease (cathepsin B, L, and S), and muscle proteases (lysosomal cathepsin, alkaline, and neutral protease) has been used to hydrolyze marine proteins [78,79]. However, acidic proteases have a lesser role in autolysis because most studies have shown that hydrolysis by endogenous proteases usually occurs at around neutral pH [2] unless this group of proteases is first isolated. Then, their effects are investigated in acidic pH [80,81,82], although the cost associated with purification of viscera proteases, their stability and activity may be a challenge compared to highly stable commercial enzymes. With autolysis, several variables, such as the freshness of by-products, may affect the optimal proteolytic activity and the possible denaturation of endogenous enzymes due to the improper handling and storage of by-products affecting the autolysis efficiency and, thus, the end products’ properties. However, because autolysis is not part of the goals of this review, more detailed information on autolytic hydrolysis technology can be found in previous studies [2]. In general, to obtain protein hydrolysates with stable peptide profiles, smell, nutritional value, functional properties, biological activities, and bioavailability, enzymatic hydrolysis must be controlled at each step of the production process. Figure 1 shows the factors involved and the main issues to be considered during the enzymatic hydrolysis of fishery by-products. The properties and structure of the end products are governed by three main factors (by-products, enzymes, and operating parameters) (Section 2.1, Section 2.2 and Section 2.3).

### 2.1. By-Product Composition, Quality, Storage and Handling

The type of by-products (viscera, heads, frames, trimmings, or their mixtures) and their composition (different amounts of lipids, blood, hemoglobin, and metal ions as pro-oxidants) influence the composition, nutritional, and antioxidant properties of the protein hydrolysates [15,83]. Frames, trimmings and heads are clean by-products with potential uses for direct human consumption or as functional hydrolysates. A recent study has shown that 37.2, 56.7, and 81.0% of the weight of heads, frames, and trimmings of Atlantic salmon, respectively, are edible as direct human food while for the viscera, the edible yield is normally thought of as 0% [10], due to the presence of lipids, bile acids, blood, non-digested feed in the intestine, etc., which is associated with a high rate of oxidation and the formation of undesirable metabolites during hydrolysis [3]. According to a survey of methods to utilize Scottish salmon by-products, 15% of the final utilization (frames, trimmings, heads) is food, 75% (frames, heads, viscera, mixed by-products, skins) is for feed purposes, and 10% (blood) is for fuel and fertilizer [6]. Therefore, cleaner by-products (frames, heads, trimmings) with acceptable freshness are ideal for food purposes, including the production of protein hydrolysates, while viscera (alone) is used for producing hydrolyzed protein concentrate and oil or fish meal and oil (rendering) in the form of mixed by-products. Some of the oxidation products can add carbonyl derivatives to the peptides, decreasing their antioxidant activity and possibly their safety. In this regard, Aspevic et al. [84] reported the differences in essential AAs, biogenic amines, and sensory properties of protein hydrolysates from the backbones, heads, and viscera of salmon and mackerel. Viscera hydrolysates from salmon (SV) and mackerel (MV) had a more intense taste and bitterness compared to hydrolysates from heads and backbones. They also showed more small peptides with MW < 200 Da (64.7 and 74.5% for SV and MV, respectively) due to the activity of endogamous enzymes during enzymatic hydrolysis with FoodPro PNL (10 U/g protein, 50 min at 55 °C). The presence of bile may add to the bitter taste sensation. SV and MV had higher TMA, cadaverine and putrescine compared to hydrolysates from heads and backbones. MV had a more intense taste and had higher scores for umami, salty, and fish taste compared to salmon hydrolysates, partly due to a higher content of ash (38.3%) in mackerel head hydrolysates, indicating the need for salt removal before human use. Heads and backbones hydrolysates showed much lower free AAs than viscera hydrolysates in both species (~40 g/100 g visceral protein), indicating autolytic hydrolysis that causes higher free AAs when using viscera as the raw material for protein hydrolysates. Despite these results, viscera hydrolysates are unlikely to be suitable for use in foods due to safety issues. On the other hand, its potential as functional feed ingredients in the form of acid silage or liquid hydrolysates can be used to enhance the growth, nutrition and health at the larval stage of aquatic species [85]. However, in most studies, the purpose of hydrolyzing viscera and identifying its antioxidant peptides is not clearly stated, although it generally suggests the direct use of viscera hydrolysates (which raises safety issues) in foods or the use of synthetized peptides, for which economic viability needs to be considered.

The freshness of by-products is one factor affecting the formation of oxidative compounds during hydrolysis, and eventually the structures, functions, and shelf life of protein hydrolysates [2,3]. Endogenous muscle proteases including matrix metalloprotease (MMP), and serine and cysteine proteases degrade myofibrillar proteins and microfibrillar networks, resulting in a significant decrease in quality [86,87,88]. These proteases are active in trimmings, frames, and heads, which with inappropriate storage temperatures, degrade myofibrillar proteins, lowering the initial quality of proteins for further hydrolysis. A recent survey of fishery by-products handling practices in Europe indicated that the sorting of by-product fractions was performed in >60% of seafood processing plants, while the remainder did not handle their by-products properly and only 25% of the surveyed plants managed by-products as food-grade. According to that survey, the majority of the processing companies used their by-products for non-food purposes, mainly as feed [58]. A study on salmon by-products (frames, heads, and viscera) showed that storage at 4 or 10 °C greatly influenced the quality. Several metabolites including tyramine, histamine, and trimethylamine (TMA) were formed, and the decline in the quality of viscera increased histamine level that exceeded the limits after 2 days of storage at 10 °C, while those by-products could be stored for up to 7 days at 4 °C [57]. Therefore, by-products processing should be carried out near the fish processing centers so the hydrolysis of by-products can be performed in the shortest possible time. When the quantity of by-products exceeds the capacity of the plant, they should be stored frozen. Therefore, the impact of frozen storage for varying times needs further study on the structure of peptides (AA composition, sequence, and size) and their biological functions.

The pretreatments of by-products may lower the concentration of pro-oxidants while resulting in purer protein substrates for enzymatic hydrolysis. The rinsing or incubation of herring by-products (heads, backbone with caudal fin, skin, intestines, and eggs) with antioxidant solutions including Duralox-MANC, isoascorbic acid, isoascorbic acid + ethylenediaminetetraacetic acid) decreased the rate of lipid oxidation and hemoglobin levels during storage at 4 °C for up to 12 days while extending the shelf-life of by-products from <1 to >12 days with a rinsing strategy or to >7 days with direct additions into the by-products after mincing [62]. However, due to the high rate of lipid oxidation in such a highly sensitive system, upgrading to food grade with proper safety is a big challenge. The washing or defatting of underutilized Sind sardine muscle mince significantly decreased total pigments (600 and 140 μg/g dry sample) and heme iron (5.3 and 1.2 mg/100 g dry sample) content in washed and defatted mince substrates, respectively, compared with non-pretreated mince (2570 μg/g dry sample and 23 mg/100 g dry sample for total pigments and heme iron, respectively), resulting in the lower formation of TBARS but increased the DPPH radical scavenging and ferrous chelating activities of protein hydrolysates, especially with defatted mince [54]. The pretreatment of cape hake by-products with 8 mM CaCl_2_ + 5 mM citric acid followed by the alkaline solubilization of proteins (pH 11) resulted in significantly lower phospholipids (1 AU/g) and lipids (0.39%) but higher solubility in hydrolysates compared to samples directly produced from by-products (4.2 AU/g and 0.87%, respectively). However, these hydrolysates were characterized by a higher yellowness and redness that was attributed to the alkaline solubilization of heme proteins during protein isolation [89]. Despite the relative improvement in by-product quality, the economic issue of carrying out such pretreatments at the tonnage scale (i.e., industrially) is a challenge that has not been studied. To tackle technological problems associated with heme proteins and to lower oxidation, the use of antioxidative extracts from agricultural waste, including lingonberry press-cake; apple, oat, barley, and shrimp by-products; and seaweed (ulva) extracts as helpers, all at 30% of the dry weight of the by-product, decreased the formation of MDA and the oxidation product 4-hydroxy-(E)-2-hexenal (HHE) in herring and salmon heads and backbone protein isolates, resulting in more stable substrates. Of all the helpers, lingonberry press-cake followed by apple peel and ulva were the most effective in reducing lipid oxidation during alkaline solubilization/acid precipitation and 9 days of ice storage. The new color (dark purple) in the resulting protein isolates with lingonberry press-cake might be advantageous for increasing the acceptance of the color by consumers [90]. Further work is needed on the interactions between the phenolic compounds of plant extracts as antioxidants and the by-product proteins, and how it affects the structure and biological activity of the protein hydrolysates. 

### 2.2. Proteolytic Enzymes

The composition and sequence of peptides in whole hydrolysates from the same source of protein may differ depending on the type of enzyme used. Endopeptidases (e.g., trypsin, chymotrypsin, pepsin, pancreatin, papain, and Alcalase) act separately from the N- or C-terminus, while exopeptidases (e.g., carboxypeptidase Y, aminopeptidase M, and Flavourzyme) break peptide bonds at the terminus of polypeptide chains [91]. Since different enzymes have specific cleavage sites (papain: Arg-, Lys- and Phe-; Alcalase: Ala-, Leu-, Val-, Tyr-, Phe- and Try-; trypsin: Arg- and Lys-; pepsin: Phe- and Leu-), different cleavage sites will affect the AA composition and the sequence of peptides [92]. Salmon skin gelatin hydrolyzed with Alcalase showed a higher content of hydrophobic AAs and a degree of hydrolysis (DH), surface hydrophobicity, and peptides with MW < 1 kDa than hydrolysates produced using Neutrase, Protamex, and Flavourzyme. This was associated with significantly higher OH^•^ and O_2_^•−^ scavenging and Fe^2+^ chelating activity [42]. From tilapia skin gelatin, different peptides, including Gly-Pro-Ala [12], Glu-Gly-Leu (317 Da) and Tyr-Gly-Asp-Glu-Tyr (645 Da) [32], Asp-Pro-Ala-Leu-Ala-Thr-Glu-Pro-Asp-Pro-Met-Pro-Phe (1383 Da) [33], Leu-Ser-Gly-Tyr-Gly-Pro (592 Da) [34], and Tyr-Gly-Thr-Gly-Leu (509 Da) and Leu-Val-Phe-Leu (490 Da) [35] were obtained depending on the enzyme used despite starting with the same protein (tilapia skin gelatin (although the method of production of the gelatin may have differed)). In abalone viscera, different enzymes resulted in different peptide sequences: Alcalase—Gln-Ser-Cys-Ala-Arg-Phe (711 Da), Ala-Ala-Pro-Ala-Val-Ser-Gly-Arg (728 Da), Asn-Arg-Phe-Gly-Val-Ser-Arg (834 Da), and Pro-Val-Pro-Pro-Tyr-Lys-Ala (770 Da); Neutrase—Ala-Ala-Gln-Tyr-Ser-Arg-Asn (808 Da), Val-His-Ala-Glu-Pro-Thr-Lys (780 Da), Gly-Cys-Tyr-Val-Pro-Lys-Cys (769 Da), and Asn-Ser-His-Val-Val-Arg (711 Da); papain—Ala-Ala-Asn-Asn-Ser-Thr-Arg (732 Da), Thr-Ile-Asp-Cys-Asp-Arg (722 Da), Cys-Ile-Gly-Tyr-Asp-Arg (725 Da), Asp-Asp-Ile-Thr-Arg-Asp (734 Da), and Asp-Val-Ala-Phe-Met-Arg (738.3 Da); and trypsin—Met-Glu-Thr-Tyr (543.3 Da), Tyr-His-Gly-Phe (523 Da), Gln-Cys-Val-Arg (505 Da) [45]. Tyr-Pro-Pro-Ala-Lys (574 Da) [46] and Pro-Ile-Ile-Ser-Val-Tyr-Trp-Lys (1005 Da) [47] were purified from blue mussels using Neutrase and pepsin, respectively. Despite the influence of the structure of peptides on the selectivity of the enzymes used, the high cost of commercial proteases suggests the minimal use of enzymes for hydrolysis. As with Atlantic cod, the hydrolysis of heads with a combination of papain and bromelain at a minimal concentration of 0.1% by weight of the minced heads for 1 h resulted in reasonable peptides profile in which 56% of peptides were <2 kDa, while most peptides (~33%) had MW between 1 and 2 kDa [93].

### 2.3. Operating Parameters

The temperature and pH are adjusted according to the selected proteases to ensure high hydrolytic activity. Thus, other factors, such as the water-to-by-products ratio, type of propeller, stirring rate, enzyme deactivation step, use of nitrogen gas, antioxidant addition, time, etc., should be optimized to ensure a stable end product (Table 2). 

The Abalone food muscle hydrolyzed with papain (HPP), Protamex^®^ (HP), or an animal protease (HA) for 0.5 or 4 h showed differences in physicochemical and structural properties governed by the time of the hydrolysis and enzyme type. The fluorescence emission spectra of all hydrolysates showed a redshift of 10–12 nm compared with that of control, while fluorescence intensity was higher in hydrolysates than non-hydrolyzed proteins (AFP). Hydrolysis for four hours resulted in higher intensities in HPP, HP, and HA compared to lower hydrolysis time. Among all samples, HA-4, HPP-0.5, and HPP-4 had higher absolute ζ-potential values than AFP, indicating a higher number of ionizable groups on the protein surface that inhibited the formation of protein aggregates, consistent with solubility, S-S bonds and free –SH groups [94].

De la Fuente et al. [95] reported the differences in peptides identified from salmon viscera obtained using two different stirring methods, including conventional stirring (30 min in distilled water at room temperature) and pressurized liquid extraction (PLE; 1500 psi, distilled water as the solvent, pH 7.0, 50 °C, 15 min) despite using the same protein source. For viscera subjected to PLE, 137 peptides were identified and contained several small antioxidant peptides with sequences of Gly-Pro-Pro and Gly-Ala-Ala. On the other hand, only 67 peptides were identified in the control extract. In both extracts, the MW was in the range of 0.6 to 2.6 kDa. However, the PLE extracts contained more significant amounts of small peptides. Total antioxidant capacity, measured using ORAC and TEAC, showed that PLE viscera extract had higher values (3790 and 7770 μM Trolox Eq for TEAC and ORAC, respectively) compared to the control extracts (788 and 2450 μM Trolox Eq for TEAC and ORAC, respectively).

During hydrolysis, especially at the industrial scale, the high water addition increases the production costs due to the heating and drying needed to obtain a powder or a concentrated liquid [3]. Using less water during hydrolysis can be beneficial if it does not affect other processes and efficiency. For cod head hydrolysates with different water ratios (1:1, 1:0.75, and 1:0.5 kg/kg), the ratio was found to have little effect on hydrolysis yield, protein content, and MW distribution of peptides and, thus, high water addition might be unnecessary. In addition, the hydrolysis reaction time of 1 h was suitable to obtain hydrolysates with desirable properties [93].

Lipid oxidation is one of the main challenges during the enzymatic/autolytic hydrolysis of by-products, resulting in unpleasant odors and flavors, dark colorations, and the formation of oxidative products in the FPH [2,3]. Due to the low lipid content of heads (1–4%), a separated oil faction was not formed in the cod head hydrolysates (0.65% lipids in the FPH) [93]. When working with a mixture of cod viscera and trimmings, a separate oil faction was formed after enzymatic hydrolysis, and a minimum of 6 g of lipids/100 g wet weight by-products was required to form an oil fraction [96]. The intensity of lipid oxidation during hydrolysis is different among by-products with different compositions, which may affect the structure and safety of the resulting peptides and their antioxidant activity. Reduction in oxygen and replacing it with an inert gas such as nitrogen decreased lipid oxidation in Sind sardine [54] and tuna [97] protein hydrolysates. 

Enzyme deactivation is the last step of the hydrolysis process. The high temperature (80–100 °C, 10–15 min) is often used to deactivate enzymes [11,12,13,14,15,16]. This temperature may lead to structural changes in the protein hydrolysates and peptides, decreasing antioxidant or other bioactivities. Xie et al. [98] used slow (+4 °C) and rapid (−18 °C) cold deactivation temperatures, a 100 °C water bath, and no deactivation. They found that the deactivation method significantly affected the DH, surface hydrophobicity, average particle size, intrinsic fluorescence, secondary structure content (α-helix, β-sheet, β-turns, and random coils), and antioxidant activity. 

The storage of protein hydrolysates as a powder following spray-drying may affect their function and properties due to hygroscopicity. If stored at temperatures above the glass transition temperature (T_g_), protein hydrolysates will be sticky (10–20 °C above T_g_), will cake (20–30 °C above T_g_), and will collapse (40–50 °C above T_g_). Thus, to avoid the loss of quality and function, powders must be stored at temperatures below their T_g_ [40].

**Table 2:** Enzymatic hydrolysis and characteristics of protein hydrolysates from fishery by-products.

**Species**	**By-Products**	**Enzymes**	**Hydrolysis Conditions**	Characteristics	**Suggested Applications**	References
Channel catfish (*Ictalurus punctatus*)	Heads + frames (3:2 *w/w*)	Papain, ficin, bromelain, neutrase, Alcalase, Protamex, novo-proD, and thermolysin	Enzymes concentrations: 10–80 AzU/g of protein in substrate; Hydrolysis time: 10–120 min; Temperature: 40–70 °C (for thermolysin) and 30–60 °C for all other proteases; pH: 7.2	The highest DH (71%) was obtained with ficin (80 AzU/g, 120 min, 30 °C), hydrolysates from novo-proD (5 and 25 AzU/g, 10 and 20 min) and thermolysin (25 AzU/g, 20 min) at 30 and 60 °C showed comparable emulsion activity index (EAI) and emulsion stability index (ESI) as soy protein isolate (SPI)	Alternative to soy or other proteins	[99]
Hake (*Merluccius merluccius*)	Undersize (discards)	A: endopeptidase of the serine type; P: broad-spectrum endopeptidase; T: trypsin-specific protease; C: chymotrypsin-specific protease; G: glutamic acid-specific protease, P + G	A: 1%, 50–70 °C, pH 6–9; P: 1%, 50 °C, pH 6; T: 1%, 45 °C, pH 6; C: 1%, 70 °C, pH 6; G: 1%, 50 °C, pH 6; P + G: 1% of each enzyme, 50 °C, pH 6	Protein extraction yield: 68%, average; MW: 2.5 kDa, antioxidant activity: 88.5 mg TE/g protein obtained with endopeptidase of the serine type (A)	Food ingredient	[100]
Smooth hound (*Mustelus mustelus*)	Viscera	Neutrase^®^, Esperase^®^, Purafect^®^, endogenous enzymes	Purafect: pH 10.0, 50 °C; Esperase: pH 9.0, 50 °C; Neutrase: pH 7.0, 50 °C; autolysis: pH 8.0, 50 °C	The DH values of 30, 27.1, 14.2, and 6.8 were obtained using Purafect, Esperase, Endogenous enzymes, and Neutrase, respectively. Ultrafiltration (UF) fraction with MW < 5 kDa from Purafect hydrolysates showed the highest antioxidant and antihypertensive activities	Functional foods	[101]
Tilapia (*Oreochromis niloticus*)	Frames	Properase E, pepsin, trypsin, flavourzyme, neutrase, gc106, papain	Properase E: pH 9, 50 °C, 4 h, E/S: 1:50; Pepsin: pH 2, 37 °C, 6 h, E/S: 1:50; Trypsin: pH 7.5, 45 °C, 3 h, E/S: 1:100; Flavourzyme: pH 7, 45 °C, 4 h, E/S: 1:100; Neutrase: pH 7, 45 °C, 4 h, E/S: 1:50 Gc106: pH 4.5, 45 °C, 6 h, E/S: 1:33; Papain: pH 6, 37 °C, 3 h, E/S: 1:100	DH: 3.8–15.1; DPPH RSA: 26–70%; ^•^OH RSA: 23.7–89%; ^•^O_2_ RSA: 1.5–58.5%; H_2_O_2_ RSA: 29–72%	Functional foods	[102]
Anchovy (*Engraulis encrasicolus*)	Viscera	Combined Alcalase, Flavourzyme, and Protamex at a 1.1:1.0:0:9 ratio	Temperature: 50 °C pH: 7.5; Stirring: 150 rpm; Time: 3 h; E:S ration: 3% (*w/w*)	Glutamic cid, glycine, alanine, and lysine comprising 11.8, 10.9, 12 and 10.9 g/100 g of hydrolysates; Heavy metals (mg/kg); Cd: 0.04 Pb: 0.25 Hg: 0.02	Nutraceuticals	[103,104]
Bigeye tuna (*Thunnus obesus*)	Mixture of heads, fins, and backbone	Pepsin	Enzyme concentration: 0.1 g/100 g waste mince; Hydrolysis time: 5 h; Temperature: 37 °C pH: 2.0	Protein: 76.4%; Lipid: 10.8%; Ash: 12.2%; Moisture: 2%; Yield: 11%; DH: 39%; EAA: 25%; NEAA: 10.3%;	Aquafeed	[105]
Rainbow trout (*Oncorhynchus mykiss*)	Mixture of heads, frames, and viscera	Endogenous enzymes (autolysis)	Time: 1–3 h; Temperature: 40–60 °C; pH: 7.1 (original pH of the by-products, no pH adjustment)	Peptide < 1 kDa: 93.2% at 40 °C for 1 h of autolysis; DPPH: 2.7–4.1 µM; TE/g hydrolysate HRSA: 81–98.2%; Metal chelating: 6.2–28.5 µM; EDTA/g hydrolysate	Food/feed ingredients	[59]
Red tilapia (*Oreochromis* spp.)	Viscera	Alcalase	E:S ratio: 1:10 (*w/w*); Temperature: 59 °C; Protein concentration: 10 g/L; Stirring speed: 51 rpm; Time: 3 h	Protein: 42.2%; Lipid: 3.6%; Ash: 22.9%; EAA: 349 residue/1000 residues; HAA: 387 residue/1000 residues; Peptides with MW of 336 Da were the main fractions; ABTS RSA: 536 μM TE/g; FRAP: 115 μM TE/g; Chelation of Fe^2+^: 377 μM EDTA/g	Food applications	[106,107]
Red tilapia (*Oreochromis* spp.)	Viscera	Alcalase	Optimal conditions: E:S ratio: 0.306 U/g; Substarte concentration: 8 g protein/L; Time: 3 h; Temperature: 60 °C; pH: 10	DH: 42.5% Iron-binding capacity of hydrolysate (RTVH-B): 67.1%; Iron-binding capacity of <1 kDa UF fraction (FRTVH-V): 95.8%	Dietary supplements to improve iron absorption	[108]
Monkfish (*Lophius piscatorius*)	Heads, viscera	Alcalase	Optimal conditions: E:S ratio: 0.05% (*v/w*); Time: 3 h; Temperature: 57 °C; pH: 8.3; Stirring rate: 200 rpm	Head hydrolysate: Protein: 69.8%; Lipid: 2.4%; Ash: 18.5%; Moisture: 9.3%; Peptides < 1 kDa: 54.6%; DPPH RSA: 45%; ABTS RSA: 13.5 μg BHT/mL. Viscera hydrolysate: Protein: 67.4%; Lipid: 4.8%; Ash: 19.7%; Moisture: 5.2%; Peptides < 1 kDa: 73.7%; DPPH RSA: 49.7%; ABTS RSA: 14.5 μg BHT/mL	Protein-rich ingredient for food or feed applications	[109]
Atlantic salmon (*Salmo salar*)	Heads, trimmings, frames	Alcalase	Optimal conditions: E:S ratio: 0.2% (*v/w*); Time: 3 h; Temperature: 64 °C; pH: 9.0; Stirring rate: 200 rpm; Solid:liquid: 1:1	Head hydrolysate: Protein: 64.2%; Peptides < 1 kDa: 33%; Digestability: 93%; DPPH RSA: 45.3%; ABTS RSA: 13.1 μg BHT/mL. Frames + trimmings hydrolysate (S-TF): Protein: 71.1%; Peptides < 1 kDa: 48.4%; Digestability: 94.1%; DPPH RSA: 56.8%; ABTS RSA: 16.8 μg BHT/mL	Aquafeed	[16]
Gurnard (*Trigla* spp.)	Heads, skin + bone	Alcalase	Concentration: 2.5 mL/kg by-products; Time: 3 h; Temperature: 61 °C; pH: 8.6; Stirring rate: 200 rpm	Head hydrolysate: DH: 24–27%; Average MW: 1379–1626 Da; Total soluble protein: 88–94.8 g/L. Skin + bone hydrolysate: DH: 19–24%; Average MW: 1203–1562 Da; Total soluble protein: 81.9–89.2 g/L	Food and nutraceutical ingredient	[83]
Blue Whiting (*Micromesistius poutassou*)	Undersized fish	Food-grade protease of microbial origin	Fish:water ratio: 1.7–2:1; Time: 45–120 min; Temperature: 50 °C;	DH: 27–45%; Protein: 70–74%; Lipid < 0.5%; Peptides < 1 kDa: 55–78%	Anti-diabetic related functional ingredients	[110]
Sprat (*Sprattus sprattus*)	Undersized fish	Commercial SPH from BioMarine Ingredients Ireland Ltd. (Monaghan, Ireland A75 WR82, IE)	Simulated gastrointestinal digestion (SGID): Pepsin E:S ratio: 2.5% (*w/w*), pH 2 for 90 min at 37 °C; Pancreatin E:S ratio: 1% (*w/w*), pH 7 for 150 min at 37 °C	DH: 39.7%; Peptide < 1 kDa: 88.7%; EAA: 335.9; NEAA: 498.3; TAA: 834.2; Solubility > 90%; over pH range of 2–12; ORAC: 588 μM TE/g sample; FRAP: 10.9 μM TE/g sample.	Promote muscle enhancement	[111]
Pacific white shrimp (*Litopenaeus vannamei*)	Shells and heads	Papain	Box–Behnken design (BBD) optimization: Temperature: 45–55 °C; pH: 6.5–7.5; Time: 30–90 min; E/S (%): 1–2	DH: 46–57%; Protein: 86.2%; DPPH: 89.6%; FRAP: 2230 μmol TE/mL. CPSS: DH: 47–54%; Protein: 83.3%; DPPH: 79%; FRAP: 1380 μmol TE/mL	As a nutraceutical in the food industry	[112]

### 2.4. Process Scale-Up

One roadblock to the industrial production of protein hydrolysates from by-products is that most studies with by-products were performed at the laboratory scale, which limits their industrial adaptation [113]. Some studies attempted pilot trials to confirm the technical feasibility of the laboratory scale at an industrial scale. In this sense, the process of producing peptides from hake by-catches was scaled up from a 0.5 to a 150 L reactor using the optimized hydrolysis conditions (2% enzyme, two h, 50% solids, pH 9, 70 °C) that were identified in the laboratory [100]. The authors found similar results at the pilot plant scale in terms of protein extraction yield (60.0% pilot and 61.4% lab), antioxidant capacity (172 mg TEAC/g protein in pilot and 224 mg TEAC/g protein in lab), and antioxidant capacity yield (103 mg TEAC/g protein in pilot and 132 mg TEAC/g protein in lab). Furthermore, liquid, solid, and bone yield did not show any significant differences from the results of the laboratory trials. Monkfish by-products (heads and viscera) hydrolysis was scaled up from 100 mL to a 5 L glass reactor at optimized laboratory conditions: 57 °C, pH 8.3, solid to liquid (S/L) ratio of 1:1 (*w*/*w*), 0.05% Alcalase, and a 200 rpm stirring rate for three hours. Following hydrolysis, the hydrolysates were filtered (100 μm) to remove non-hydrolyzed materials. Results validated the properties of the FPH obtained by the optimization trial at the laboratory scale. However, the 5 L reactor may still be considered a bench-scale trial and needs work with larger reactors [109]. The industrial-scale production of hydrolysates from Atlantic salmon by-products (heads, frames, and viscera) using the laboratory (4 L glass vessel) hydrolysis parameters such as enzyme type and concentrations, time and temperature, except for a shorter time from slaughter to hydrolysis (2 h), the avoidance of preheating the water inside the reactor, separation using a decanter, and no drying of the hydrolysis solution (acidified with formic acid to reach pH < 4) was tested in an industrial plant. The authors attributed the main differences to lower hydrolysis efficiency, separation, and storage conditions [15]. Overall, the cost of enzymes, high water usage, and the drying or condensation of the hydrolysis solution, as well as the use of separators (one or more steps) to remove oil and other undigested materials are the main causes of the greater process costs at an industrial scale. However, the complexity of the hydrolysis line depends on the type and composition of the by-products. 

## 3. Antioxidant Activity of Fishery By-Products Protein Hydrolysates and Peptides

The antioxidant peptides had 2–10 amino acids, although some had up to 20 amino acids and had a MW of 0.2 to 2 kDa. The antioxidant activity of peptides is mainly related to the presence and position of specific amino acid residues in the peptide chain. Primary structure, amino acid composition, hydrophobicity, spatial conformation, etc., are characteristics that are affected by enzymatic hydrolysis and determine its antioxidant activity [114]. Peptides show antioxidant activity due to the presence of one or more hydrophobic (Pro, Ala, Gly, Leu, Ile, Met, Trp, Phe, Val) and aromatic (Tyr, Trp, Phe) amino acids that can quench free radicals via various mechanisms, including hydrogen atom transfer (HAT), single electron transfer followed by proton transfer (SET-PT), and sequential proton loss by electron transfer (SPLET) mechanisms [115]. Scavenging free radicals and oxidants using HAT, SET-PT, and SPLET generally leads to the same end results, although the kinetics and potential for side reactions vary. SPLET, SET-PT, and HAT may occur in parallel; however, the dominant mechanism depends on the antioxidant’s conformational and geometrical features, solubility, partition coefficient, and the type of solvents [116]. The antioxidant activity of hydrophobic amino acids has been attributed to their ability to interact with lipid molecules by increasing the solubility of peptides in lipids and scavenging lipid-derived radicals through electron-donating substituents such as OH and NH_2_ on amino acid side chains in peptides. Hydrophobic amino acids can improve the antioxidant activity of peptides by providing a potential pool of free electrons. Additionally, it is believed that aromatic amino acid residues might possess antioxidant properties due to the chelating ability of the imidazole ring and the trapping ability of lipids [115,116].

Peptides containing proline-rich sequences have been identified to possess antioxidant properties. Proline has an electron-rich nitrogen-containing pyrrolidone ring that stabilizes the radical peptide formed after electron donation [117,118]. Peptides with Pro at the C-terminus (e.g., Pox: Tyr-Tyr-His-Pro) were the most potent antioxidant (0.8 TE at 2.5 μM). The modification of the structure by moving Pro into positions X1 (Pro-Tyr-Tyr-His), X2 (Tyr-Pro-Tyr-His), and X3 (Tyr-Tyr-Pro-His), but leaving the other residues in the same order as in Pox, resulted in a significant difference in ORAC, which was 0.2, 0.1, and 0.55 TE at 2.5 μM for X1, X2, and X3, respectively [119]. Intense antioxidant activity has been reported for small peptides containing amino acid residues such as Tyr, His, and Pro [120,121]. The dipeptide Tyr-Tyr at the N-terminal position of Tyr-Tyr-His-Pro and Tyr-Tyr-Pro-His was the portion responsible for stronger antioxidant activity. However, Tyr-Pro-Tyr-His showed the weakest ORAC and inhibited ROS production by 36% at 0.07 μM in human keratinocyte cells (HaCat) after treatment with H_2_O_2_ when compared to Tyr-Tyr-His-Pro (with the highest ORAC), which showed the similar inhibition of ROS production at 2.5 μM (40%) [119]. It is believed that besides amino acid composition and sequence, the changes in secondary structure have a significant impact on the capture and dissipation of free radicals. The nanopeptide Val-Leu-Leu-Tyr-Lys-Asp-His-Cys-His (1127 Da) produced using the self-assembly of pine nut Val-Leu-Leu-Tyr (506 Da) and sea cucumber Lys-Asp-His-Cys-His (638 Da) had significantly higher antioxidant activity compared to individual peptides due to changes in the secondary structure as seen in the lower electron paramagnetic resonance (EPR) signal, higher random crimp degree, and increased supply of hydrogen protons (i.e., the higher exposure to active hydrogen) from Raman spectroscopy and ^1^H NMR spectrum analysis in the nanopeptide than the tetrapeptide and the pentapeptide. The DPPH radical scavenging activities at 3 mmol/mL were 6.1, 9.4, and 80.7% of the 4, 5, and 10 amino acid peptides, respectively [122]. 

The presence of Tyr at the N-terminal position of peptide Tyr-Ala-Glu-Glu-Arg-Tyr-Pro-Ile-Leu has been reported as the residue that most contributed to antioxidant activity (3.8 μM TE/mg protein). However, Tyr-Pro-Ile and Tyr-Gln-Ile-Gly-Leu with Tyr at the same position showed lower ORAC (1.6 and 1.7 μM TE/mg protein, respectively), indicating the role of adjacent amino acids and chain length on antioxidant activity [123]. Tyr-containing peptides from abalone viscera showed strong ABTS radical scavenging activity in the order of Cys-Ile-Gly-Tyr-Asp-Arg (0.144 mg/mL) > Tyr-His-Gly-Phe (0.268 mg/mL) > and Gly-Cys-Tyr-Val-Pro-Lys-Cys (0.389 mg/mL). The first and last peptides, which contained both Tyr and Cys, showed similar trends for scavenging DPPH radicals (IC_50_ of 0.207 and 0.405 mg/mL, respectively). Despite the observed high ABTS radical scavenging activity, peptides Met-Glu-Thr-Tyr and Tyr-His-Gly-Phe, which had Tyr at the C- or N-terminal position, respectively, had weak scavenging activity against DPPH radicals (<20%), which was attributed to the lack of Cys in their sequence. Despite having different sizes or amino acids residues, peptides Gln-Cys-Val-Arg and Gln-Ser-Cys-Ala-Arg-Phe showed similar DPPH radical scavenging activity (IC_50_ of 0.392 and 0.416 mg/mL, respectively), indicating the complexity of the relationship between the peptides’ structures and function. Regarding the number of amino acid residues within peptide sequence, the peptide Gly-Cys-Tyr-Val-Pro-Lys-Cys, containing two Cys residues, showed lower free radical scavenging activity (IC_50_ of 0.389 and 0.405 mg/mL for scavenging ABTS and DHHP radicals, respectively) than Cys-Ile-Gly-Tyr-Asp-Arg, which contained only one Cys (IC_50_ of 0.144 and 0.207 mg/mL for scavenging ABTS and DPPH radicals, respectively [124]. Thus, a simple relationship between the number of Cys and antioxidant effects of peptides is not clear and is more related to its position in the peptide chain and the type of amino acids adjacent to it. Generally, the thiol group of Cys has an antioxidant activity that functions by donating hydrogen from the SH group or losing an electron from its sulfur atom, thus neutralizing free radicals [24].

It was shown that the presence of a Tyr, Trp, Cys, or Met residue with electron/hydrogen donating ability was the driving force for dipeptides to scavenge radicals. The presence of Tyr, Trp, and Cys in the sequence was required for dipeptides to scavenge ABTS^•+^, while the presence of Tyr, Trp, Cys, and Met was needed for dipeptides to scavenge ROO^•^ when using the ORAC assay. Structure–activity relationships showed that Tyr- and Trp-containing dipeptides with Tyr/Trp residue at the N-terminus (Tyr/Trp-X; Tyr-Gly, Tyr-Ser, Tyr-Gln, Tyr-Glu) had stronger ORAC and ABTS^•+^ scavenging activity than that at the C-terminus (X-Tyr/Trp, Gly-Tyr, and Glu-Tyr) and the steric effects, hydrophobicity, and hydrogen bonding also affected the neighboring AAs. Tyr-containing dipeptides showed higher ABTS^•+^ scavenging activity. In contrast, Trp dipeptides (Trp-Gly, Trp-Ser, Trp-Gln, Trp-Glu, Gly-Trp, Glu-Trp) had higher ORAC, and only Cys-containing dipeptides showed moderate reducing power activities [125]. The calculation of BDE, IP, PA, and ETE of Tyr/Trp-X and X-Tyr/Trp (where X was Gly, Leu, Pro, Phe, Ser, Thr, Asn, Gln, Asp, Glu, Lys, and Arg) showed that there were few differences among dipeptides, indicating that the neighboring AAs did not affect the intrinsic hydrogen or electron-donating ability of the dipeptides studied. Thus, the differences in their radical scavenging activity can be attributed to other factors (such as steric effects and inter/intra-molecular hydrogen bonds). Furthermore, the BDE and PA of Tyr-containing dipeptides were much lower. At the same time, its IP was higher than Trp-X or X-Trp, indicating that HAT and SPLET mechanisms were more favorable for Tyr-containing dipeptides, while SET-PT was the primary mechanism of antioxidant activity of Trp-containing dipeptides [125]. 

Aromatic and acidic amino acids are effective proton and electron donors to neutralize free radicals [126]. The high DPPH^•^ and OH^•^ scavenging activity of Glu-Ala-Pro-Val-Glu-Gly-Gly-Leu-Phe-Asp-Tyr-Val-Lys from scallops has been attributed to the presence of the acidic amino acids (Glu and Asp) and two aromatic amino acids (Phe and Tyr) in the sequence. At the same time, Arg at the C-terminal contributed to the high ORAC (6.18 μM TE/μmol) of Lys-Leu-Ala-Asp-Met-Leu-Asn-Pro-Glu-Arg [127]. Tyr and Phe in Gly-Glu-Tyr-Gly-Phe-Glu and Phe in Gly-Ile-Glu-Leu-Phe-Pro-Gly-Leu-Pro sturgeon cartilage contributed to higher DPPH^•^ and OH^•^ scavenging activity [128]. 

The antioxidant activity of His-containing peptides has been reported and attributed to the chelating and lipid radical-trapping ability of the imidazole ring [129]. The removal of His from the C-terminal position of Val-Asn-Ala-Val-Leu-His (MW: 651 Da) significantly decreased the DPPH (~20%) and ABTS (~16%) radical scavenging activity of the modified peptide Val-Asn-Ala-Val-Leu (MW: 514 Da) compared to ~33% and 21% for the original sequence. As seen using circular dichroism (CD) spectroscopy, the secondary structure of Val-Asn-Ala-Val-Leu had no α-helix with a low band intensity at 195 nm, probably due to the shortening of the peptide size after removing the C-terminal His. The treatment of the original and modified peptides with the 40 kV/cm pulsed electric field (PEF) transferred the β-sheet to the random coil. This led to higher antioxidant activity, and Val-Asn-Ala-Val-Leu-His was much more sensitive to PEF than Val-Asn-Ala-Val-Leu, which exposed the leading active site of the C-terminal His by altering the secondary structure of the peptide [121]. 

Metal-chelating peptides (MCP) can complex transition metal ions, such as Fe^2+^ and Cu^2+^, involved in ROS production using the Fenton and Haber–Weiss reactions. Therefore, peptides with metal chelating ability can act as indirect antioxidants, reducing or inhibiting food oxidation, and increasing food shelf life, while reducing the oxidation products associated with age-related diseases [91]. These peptides can also be used as supplements to provide dietary minerals such as Ca, Zn, and Fe with high absorption rates [130,131,132]. His, Lys, Arg, Pro, and Gly were abundant in peptides with metal chelating ability [133]. Glu, Asp, and Gly residues were the major AAs in the 26 identified peptides from anchovy stick water hydrolysates. Peptides containing these AAs formed complexes with Ca ions more effectively [131]. From tilapia skin gelatin, Gly-Pro-Ala-Gly-Pro-Ala-Gly-Glu-Lys (782 Da), Asp-Gly-Pro-Ser-Gly-Pro-Lys-Gly-Asp-Arg (984 Da), Gly-Leu-Pro-Gly-Pro-Ser-Gly-Glu-Glu-Gly-Lys-Arg (1198 Da), and Asp-Gly-Pro-Ser-Gly-Pro-Lys-Gly-Asp-Arg-Gly-Glu-Thr-Gly-Leu (1441 Da) have been purified from trypsin hydrolysates and showed high Fe^2+^ chelating capacity. Each peptide contained one or more acidic amino acids, i.e., Glu and Asp, or basic amino acids such as Lys and Arg. In addition, three of the four Fe^2+^-chelating peptides contained Ser [130]. Asp, Glu, Gly, and Pro were the primary amino acids in sea cucumber metal-chelating peptides [132].

## 4. Application of Fish By-Products Protein Hydrolysates to Control Oxidative Deteriorations of Seafood

The oxidation of lipids is often the major cause of the quality loss of foods during storage, as seen in the changes in color, texture, flavor, and aroma, which impairs sensory and nutritional properties and the shelf-life of foods [28]. The decomposition of the hydroperoxides formed by pro-oxidative metal ions is a driving factor for lipid oxidation, producing highly reactive alkoxyl lipid radicals and hydroxyl ions. Alkoxyl radicals degrade rapidly to form volatile decomposition products, often with off-odors [29]. Furthermore, protein carbonyls can be introduced into proteins using a covalent linkage of lipid carbonyls (e.g., protein-bound malondialdehyde). Protein oxidation leads to functional property changes such as decreased solubility, digestibility, and water-holding capacity [30]. The loss of nutrients and myofibrillar water and the changes in texture are inevitable during frozen storage [133,134]. The formation of ice crystals, associated with cell membrane rupture and muscle fibers, often leads to protein denaturation and undesirable reactions such as aggregation and decrease in solubility, solute concentration (macromolecular crowding), lipid oxidation, and instability of proteins at the ice–water interphase [135,136]. Protein hydrolysates and peptides may be potential antioxidants to reduce oxidation during food storage, thus extending the shelf life [28]. The antioxidant activity of protein hydrolysates was related to amino acid composition, sequence, size, and the amino acid residues at the C- or N-terminal positions [114]. Enzymatic hydrolysis disrupts the tertiary structure of food proteins, leading to the increase in the solvent accessibility of peptides to scavenge free radicals and chelate pro-oxidative metal ions. Protein hydrolysates and peptides have been reported to control food oxidation through various mechanisms, including inactivating ROS, scavenging free radicals, chelating pro-oxidative metal ions, reducing lipid hydroperoxides, and changing the physical state of foods. At the same time, peptides control the formation of ice crystals and decrease protein oxidation and denaturation during storage, thus showing bi-functional effects in foods, i.e., antioxidant and cryoprotective activities [25,137]. Table 3 shows the effects of peptides and protein hydrolysates from marine by-product sources on the inhibition of/reduction in the oxidation of seafood lipids and proteins during processing or storage.

## 5. Conclusions and Future Challenges Facing By-Product Upgrades

Marine by-products have been studied as a source of antioxidant peptides for food, feed, and nutraceutical applications. Those studies recommended these peptides as potential functional ingredients to enhance health and nutrition. However, the differences in the composition of by-products, the type of proteases used, and the different hydrolysis parameters resulted in various end products from the same protein. To ensure consistency, the process of upgrading at three levels, i.e., by-products, enzymes, and operating parameters must be optimized for each by-product source. 

Different peptides were produced by different enzymes. However, it has not been determined which peptide is a more potent antioxidant in controlling oxidation in which food system. Different foods, due to inherent composition differences (i.e., different amounts of pro-oxidants, oxidation-prone substances, and internal antioxidant enzymes), will probably have different reactions to antioxidant peptides and specific structures during storage. Therefore, the effect of peptides with specific structures or protein hydrolysates produced using a specific condition in different food matrices needs to be investigated. 

Although the structure of peptides was influenced by the specificity of the proteases used, most studies used fresh by-products with acceptable initial quality. It is less known whether the same peptides structure and function can be obtained using previously stored by-products and how the quality of proteins in refrigerated or frozen by-products, as well as associated chemical reactions during storage, affect hydrolysis and product structure, function, and stability. This area needs to be further investigated, especially in by-products with high lipid and blood contents, such as herring and salmonid by-products, and to understand which fractions are more oxidized and contribute more significantly to undesirable biochemical reactions during hydrolysis. Several researchers have tried to stabilize by-products before upgrading them using antioxidants. There is a price to adding synthetic antioxidants or to maintaining the initial quality and safety of agricultural wastes as sources of antioxidative extracts. The practical ability to undertake this process for large quantities of by-products and the space and energy consumption required to create low temperature storage are among the issues that make this application more complicated.

Few studies have investigated the relationship between the initial microbial, the chemical quality of by-products, and the safety of the resulting protein hydrolysates for food applications. Residual antibiotics in protein hydrolysates from intensive fish/shrimp farming, the amount of biogenic amines such as histamine, and the presence of contaminants (such as cadmium, arsenic, mercury, and lead) caused by the pollution of the sea or culture water are safety issues that should be considered when selecting by-products. 

By-product processing should be carried out near fish production and processing centers so the hydrolysis of by-products can be performed within the shortest possible time. When the quantity of by-products exceeds a center’s capacity, they should be stored frozen. Therefore, the impact of frozen storage at varying lengths of time on the structure of peptides (amino acid composition, sequence, and size) and the occurrence of undesirable oxidative deteriorations and biochemical changes that may affect biological functions needs further study. There is limited information about the effect of initial protein quality due to processing and storage on the functional and biological activities of hydrolysates and peptides for food applications. Maintaining the consistency of the raw material properties (in terms of composition, freshness, and storage) will likely result in a consistent end product from each production batch.

Many studies are underway regarding the use of fish protein hydrolysates in food. Nevertheless, the supply of fresh raw materials with acceptable safety, competitive prices with other commercial ingredients from plants and other sources, and the lack of efficient and standardized techniques to transform fish by-products into marketable forms limit their utilization. 

Although the role of protein hydrolysates in maintaining the quality of seafood products has been shown, a standard method for its production from a specific source of marine by-products on a pilot or an industrial scale and its industrial application has not yet been undertaken. However, the industry prefers to use synthetic preservatives with lower prices to maintain the quality of seafood products during storage. As an example, in the case of shrimp processing, the suggested use of hydrolysates to soak shrimp (as whole or peeled) for one hour (compared to only a few min in the case of sulfate additives) to ensure water holding capacity and protein quality is not practical at an industrial scale for shrimp processors. They cannot wait for such a long time to process several tonnes of shrimp that come to a plant each day and must be processed within a minimum length of time.

Protein hydrolysates may also affect the sensory characteristics of food. So, how to mask or remove the fish smell using encapsulating methods and the cost of such pretreatments on protein hydrolysates and the market must be addressed. Furthermore, hygroscopicity, the development of bitterness during enzymatic hydrolysis, low storage stability, and hydrolysis in the gastrointestinal tract (GIT) may pose several challenges to the application of fish protein hydrolysates in the food industry [155,156]. Several encapsulating techniques, including liposomes, nanohydrogels, emulsions, and diphasic gel double emulsions, have been used to improve the storage and gastrointestinal stability of protein hydrolysates [157,158]. To reduce the bitterness and gastric instability and the loss in antioxidant activity, the effectiveness of each method should be considered for each particular source of marine by-product protein hydrolysates. 

## Figures and Tables

**Table 1:** Composition and main safety issues in by-products from different categories of aquatic species.

**Species**	**Yield (%) of By-Products in Relation to Whole Weight**	**By-Products Fractions and Yield (%)**	**Proximate Composition (%)**	**Main Safety Issues**	**Preventive Measures**	**References**
Fish						
Atlantic salmon (*Salmo salar*)	43.8	Heads (9.9), viscera (10.6), frames (10.4), trimmings (8.2), skin + scale (4.2)	Heads: moisture (53.2), ash (5.01), protein (17.2), lipid (21.5) Frames: moisture (52.9), ash (6.5), protein (19.3), lipid (17.16) Trimmings: moisture (46.4), ash (2.2), protein (18.1), lipid (26.4) Viscera: moisture (46.5), ash (0.97), protein (12.4), lipid (37)	Fast decomposition by endogenous proteolysis, gills and viscera blood, gall bladder, off-odor of viscera, formation of harmful compounds such as biogenic amines and trimethylamine (TMA), loss of freshness	Lowering storage time and temperature; sorting heads, frames from viscera; removal of gills where possible	[10,15,56,57]
Rainbow trout (*Oncorhynchus mykiss*)	20–30	Heads (11.2), viscera (7.8), frames (7.6), skin (3.4)	Heads: moisture (69.6) organic matter (27.7), ash (2.7) Trimmings + Frames: moisture (66.5) organic matter (30.6), ash (3)	Fast decomposition by endogenous proteolysis (autolysis), high abdominal fats, blood (gills), non-digested feed in the stomach, digesta and fecal matter in the intestines	Cold or frozen storage of by-products, sorting heads and viscera, maintaining freshness, where possible removal of gills with large amounts of blood and hemoglobin (e.g., in large fish > 2 kg), pretreatment with antioxidants	[16,58,59]
Yellowfin Tuna (*Thunnus albacares*)	50–55	Heads (13), fins (1), skin (10), bone (6), viscera (8), dark meat	Upper half: moisture (68.2), organic matter (26.1), ash (5.4) Lower half: moisture (60.6), organic matter (25.1), ash (14)	Long traveling times post-catch to processing plant; degradation and off-odor of viscera; spoilage of gills, tongue, and head flesh; huge blood release from large gills; activation of muscle proteases of heads (autolysis of proteins); contamination by heavy metals (e.g., cadmium)	Using fresh by-products (caught by longline), removal of gills (blood), cold or frozen storage of by-product before hydrolysis, sorting of viscera from heads, reject batch with high heavy metals	[17]
Turbot (*Scophthalmus maximus*)	69.6	Heads (19.6), frames (16.4) trimmings (13.5), viscera (14.3), skin + scale (5.8)	Heads: moisture (71.3), ash (6.4), protein (20.2), lipid (1.7) Frames: moisture (58.7), ash (7.9), protein (19.1), lipid (12.1) Trimmings: moisture (70.1), ash (4.3), protein (20), lipid (4.8) Viscera: moisture (70.9), ash (1.6), protein (13.4), lipid (10.9)	Bioaccumulation of heavy metals in muscle and by-products, autolysis of by-products, long time-period from catch to delivery at the dock, low freshness, bacterial spoilage	Inhibiting quality deterioration by low storage time and temperature, sorting by-products fractions, reject batch with high heavy metals	[10,60,61]
European seabass (*Dicentrarchus labrax*)	55	Heads (21.2), frames (11.9) trimmings (7.1), viscera (7.7), skin + scale (7)	Heads: moisture (59.4), ash (10.1), protein (17.8), lipid (11.2) Frames: moisture (52.6), ash (12.4), protein (18.6), lipid (13.9) Trimmings: moisture (57.5), ash (6.9), protein (21.2), lipid (11.1) Viscera: moisture (31.9), ash (1.5), protein (14.34), lipid (39.3)	Significant amount of oil and thus high lipid oxidation during hydrolysis, addition of lipid-derived carbonyls on the forming peptides, viscera spoilage, freshness	Sorting frames and trimmings from viscera, antioxidants addition, using N_2_ to control possible oxidation with viscera	[10,19]
Gilthead seabream (*Sparus aurata*)	59.9	Heads (27.6), frames (12.4) trimmings (6), viscera (6.9), skin + scale (7)	Heads: moisture (51.9), ash (8), protein (15.3), lipid (20.3) Frames: moisture (55.6), ash (9.4), protein (19.4), lipid (13.7) Trimmings: moisture (56.5), ash (4.4), protein (22.4), lipid (13.2) Viscera: moisture (60.5), ash (2), protein (17.2), lipid (12.8)	Significant amount of oil and thus high lipid oxidation during hydrolysis, addition of lipid-derived carbonyls on the forming peptides, viscera spoilage, freshness	Sorting frames and trimmings from viscera, antioxidants addition, using N_2_ to control possible oxidation with viscera	[10,19]
Herring (*Clupea harengus*)	~60	Heads (13.7–17), backbone (12.1–24.6), belly flap (4.5–10.7), tail (1.6–4), others (including viscera, blood, roe, milt, etc., depending on catch season, 2.4–17.6)	Protein (12.8–19.2), lipids (5.8–17.6), ash (1.3–7.2), moisture (65.7–78.7)	Fast oxidative deterioration, rancidity soon after filleting, blood contamination (Hb-mediated lipid oxidation), autolysis, rapid microbial spoilage, loss of freshness (biogenic amines formation)	Low storage temperature, stabilization by antioxidants, sorting of heads (due to presence of gills and blood) with other more stable fractions	[62,63]
Crustaceans						
Shrimp (*Litopenaeus* sp., *Macrobrachium* sp., *Fenneropenaeus* sp., *Penaeus* spp.)	44–62.5	Heads (34–54), shell (7.4–7.6), tail (1.7–2.8)	Head: moisture (68–75), protein (6.6–10), lipids (2.2–7), ash (4–6) Shell and tail: moisture (58–68), protein (8–11.3), lipids (0.4–0.8), ash (8.5–13.5)	Endogenous proteases activity, polyphenoloxidase activities, appearance of melanin (black spot), soft shell, antibiotic residue, heavy metals contaminants (cadmium, arsenic, mercury, lead)	Cold chain, frozen storage, use of by-products from extensive shrimp farming (no antibiotic use), raw material safety through suppliers agreement	[3,64,65]
Crab (*Portunus* sp., *Polybius* sp., *Cancer* sp., *Eriocheir* sp., *Eriphia* sp.)	>50	Shells, liver (hepatopancrease), physiological liquid (hemolymph), legs	Moisture (50–58), protein (15–30), minerals (30–50), chitin (15–30), fat (1–10)	Heavy metals (arsenic, cadmium, lead, chromium, mercury), loss of freshness (histamine, cadaverine, etc.), autolysis mediated by endogenous proteases	Storage at low temperatures, maintaining freshness, reject batch with high heavy metals	[66,67]
Lobster (*Panulirus* spp., *Jasus* sp.)	45–80	Head (20), shell, liver, eggs, hemolymph	Protein: heads (43.5), livers (41.1), shells (29} Lipids: liver (24.3), shells (0.6) Minerals: shells (36), heads (31.6)	Heavy metals (arsenic, cadmium, lead, chromium, mercury) due to marine pollution, loss of freshness (histamine, cadaverine, etc.), autolysis mediated by endogenous proteases, TMA formation	Storage at low temperatures, maintaining freshness, reject batches with high heavy metals	[66,67,68]
Cephalopods						
Squid (*Loligo* sp., *Illux* sp.)	52	Heads and tentacles (25), fins (15), viscera (8), skins (3), pen (1)	Moisture (80) protein (18), lipids (1), ash (1)	Rapid post-mortem auto-proteolytic degradation of proteins, microbial spoilage; contamination by persistent organic pollutants (POP); high concentrations of copper, zinc, and cadmium in digestive glands	Keeping freshness, reducing the rate of chemical and microbial spoilage, cold storage, removing dark ink, reject batch contaminated with high concentrations of heavy metals	[69]
Cuttlefish (*Sepia* *officinalis*)	58	Head and tentacles (23.3), viscera (18.7), fins (8.5), skin (4.2), ink (4.6)	Moisture (64–75), protein (14.9–17.5), lipids (4.8–6.2), ash (1.7–2.0)	Inappropriate storage temperature, degradation by acid and alkaline proteolytic activity, microbial spoilage, high quantity of heavy metals such as in viscera	Maintaining freshness of by-products; minimizing enzymatic degradation and microbial spoilage, especially in viscera, preservation, and cold storage of by-products	[52,70,71,72,73]
Bivalve mollusks						
Oyster (*Pinctada fucata*), mussle (*Mytilus edulis*), cockle (*Cerastoderma edule*)	~60	Shell (60), byssus threads, extracellular fluid (containing hemolymph and extrapallial fluid (EP))	Shell: protein (2.5% on a dry weight basis)	Contamination by heavy metals, gasoline, hydrocarbons, pesticides, and microorganisms (coliforms, vibrio, salmonella, shigella, biotoxins)	Monitoring concentrations in the laboratory each season, reject batches, use of disinfected water and refrigeration, implementation of good hygiene and manufacturing practices, fishermen’s health certificates	[74,75,76]

**Table 3:** Effects of marine protein hydrolysates to control oxidative deteriorations in seafood during processing or storage.

Marine Species	By-Product	Hydrolysis Conditions	Structural Properties of FPH/Peptides	Food Products	Storage Conditions	Oxidation Inhibition/Quality Preservation	References
Amur sturgeon (*Acipenser schrenckii*)	Skin	Gelatin was hydrolyzed with Alcalase (5% *w*/*w*, pH 8.0, 50 °C) for 3 h	Pro-Ala-Gly-Tyr (405 Da)	Japanese seabass mince	Six freezing (−18 °C) and thawing (+4 °C) cycles	Peptide maintained intra-myofibrillar water (T_21_) pool and reduced free water (T_22_) population, preserved thermal properties of myosin and actin, and lowered TBARS formation	[11]
Tilapia (*Oreochromis niloticus*)	Skin	Collagen was hydrolyzed with Alcalase at 4000 U/g and 60 °C for 3 h to obtain tilapia skin collagen peptide (TSCP)	Peptides with MW < 2.5 kDa accounted for 57.1% of TSCP, Gly accounted for 20.2% of amino acids, and the hydrophilic amino acids content was 38.3%. The active peptide was Asn-His-Gly-Lys (454 Da)	Scallop adductor muscles	−18 °C for 2 week	Frozen scallop muscles treated with 3 g/100 g TSCP showed higher salt soluble protein concentration, total sulfhydryl content, Ca^2+^-ATPase activity, and water-holding capacity during the 8 week storage period	[138]
Squid (*Loligo opalescens*)	Skin	Squid skin collagen was hydrolyzed with acid protease at 6000 U/g at 40 °C for 3 h to obtain collagen hydrolysates from squid skin (CH-SS)	82.3% peptides had MW < 5000 Da, among them 22.69% had MW between 1–2 kDa; Asp-Val-Arg-Gly-Ala-Glu-Gly-Ser-Ala-Gly-Leu rich in Gly tripeptide repeat sequence was identified as the active peptide	Shrimp muscle	Fourteen freezing (−25 °C) and thawing (+4 °C) cycles	CH-SS reduced the mechanical injury caused by ice crystals to shrimp muscle as well as carbonyl formation, maintaining the integrity of fiber structure, thereby reducing drip loss, higher content of α-helix, and lower random coil compared to untreated muscle	[139]
Threadfin bream (*Nemipterus hexodon*)	Skin	Skin gelatin was hydrolyzed with lizardfish pepsin (pH 2.0 and 40 ◦C) and papain (pH 7.0 and 40 ◦C) for 60 min	Gelatin hydrolysates had a DH of 10, 20, 30, and 40%	Natural actomyosin (NAM)	NAM with 8% gelatin hydrolysates was subjected to six freeze-thaw cycles (20 h freezing at −18 °C and 4 h thawing at 4 °C for each cycle)	NAM with 20% DH gelatin hydrolysates showed the highest Ca^2+^-ATPase activity, total sulfhydryl groups and solubility along with lower disulfide bond content and TBARS	[140]
Silver carp (*Hypophthalmichthys molitrix*)	Muscle	Muscle homogenate was hydrolyzed with Protamex (1.5 AU/g) for 30 min at 50 °C	DH of the hydrolysates was 13.6	Unwashed surimi	Storage at conventional (−18 °C) or ultra-low (−60 °C) temperatures and subject to three and six freeze–thaw cycles (per cycle −18/−60 °C, 12 h; 4 °C, 12 h)	Hydrolysates reduce the formation of carbonyls, TBARS, and volatiles hexanal, nonanal, and 1-octen-3-ol, while maintained total sulfhydryl group samples stored at −18 °C showed lower lipid and protein oxidation levels than those of samples stored at −60 °C, indicating structural deterioration of surimi under ultra-low frozen temperature storage	[141]
Silver carp	Meat leftovers on bones and heads	Defatted ground mince (5-folds isopropanol at 25 °C for 1 h) was hydrolyzed with Alcalase (AH; 3000 U/g; pH 8, 60 °C) or Protamex (PH; 2400 U/g, pH 7, 50 °C) for 30 min	DH for Alcalase and Protamex hydrolysates was 12.9 and 13.2% respectively, and peptides with MW of <138, 286–780, and ~1420 Da accounted for 3.5, 39.1, and 50.6% in Alcalase hydrolysates and 4.7, 16.8, and 37.4% in Protamex hydrolysates	Surimi	Surimi with 2, 4, and 6% hydrolysates subjected to six freeze–thaw cycles (−25 ± 1 °C for 12 h and 4 ± 1 °C for 12 h per cycle)	Surimi with the addition of 2 g of Protamex hydrolysate displayed the highest actomyosin extractability, Ca^2+^-ATPase activity and correspondingly, the lowest surface hydrophobicity of actomyosin, while maintaining total sulfhydryl groups and texture of heat-set gel	[142]
Large yellow croaker (*Pseudosciaena crocea*)	Muscle	Lyophilized protein (0.02 mg/mL) was hydrolyzed with pepsin (pH 2, 40 °C), trypsin (pH 8, 45 °C, and neutral protease (pH 7, 50 °C) at 5000 U/g for 5 h	Peptides with MW < 500 Da comprised 77.8, 78.5, and 74.3% of pepsin, trypsin, and neutral protease hydrolysates, respectively, and trypsin hydrolysates showed the highest content of hydrophilic amino acids (51.87%) compared to pepsin (47.26%) and neutral protease (39.14%) hydrolysates	Turbot fillets	Fillets were soaked in 2 mg/mL hydrolysate alone or in combination with 4% sucrose) for 4 h, then subjected to 3 freeze–thaw cycles (−20 °C for 24 h and 4 °C for 12 h for each cycle)	Trypsin hydrolysates reduced the loss of Ca^2+^-ATP enzyme activity and the structural integrity damage of myofibrillar protein better than other hydrolysates	[143]
Argentine croaker (*Umbrina canosai*)	Muscle	Alkali-solubilized protein was hydrolyzed with Alcalase (pH 8, 50 °C) or Protamex (pH 7, 50 °C) at 30 U/g until a DH of 20%	MW of Alcalase and Protamex hydrolysates were 1083 and 1350 Da, respectively	Flounder fillets	Fillets coated with agar film containing Alcalase hydrolysates were stored at 5 °C for 15 days	Agar-hydrolysate film showed higher transparency and mechanical properties than clove essential oil film. It improved the biochemical and microbiological qualities of fillets without the sensory limitation of the essential oil volatile compounds	[144]
Common carp (*Cyprinus carpio*)	Skin	Gelatin was hydrolyzed by 2% (proten basis) Protamex^®^ at pH 7 and 50 °C for 3 h	DPPH RSA, the metal chelating ability, and the FRAP of gelatin hydrolysates were 23.8%, 64%, and 2.65 μM TE/mg sample, respectively, and dipeptide Ala-Tyr (MW: 252 Da) was isolated as an active antioxidant peptide with FRAP of 89.3 μM TE/mg sample	Atlantic mackerel fillets	−18 °C/4 month	Ala-Tyr peptide layer on the furcellaran/gelatin hydrolysate (FUR/HGEL) films increased antioxidant activity and mechanical and rheological properties, while reducing the water solubility of the films; the reduction in fillet oxidation was not significant, and TVB-N formation was inhibited by the film	[145,146]
Blue whiting (*Micromesistius poutassou*)	Discarded material	Hydrolysis by trypsin (0.1% E:S) at pH 8 and 50 °C until reaching a DH of 4%	EC_50_ values of DPPH RSA, reducing and chelating power were 1.46, 11, and 0.95 mg/mL, respectively. BPH contained 60% of peptides between 0.5 and 3 kDa. Protein, lipid, ash, and moisture were 76.8, 9.4, 7.3, and 3.35%, respectively	Omega-3 emulsion from refined commercial fish oil (18% EPA and a 12% DHA)	20 °C/10 days	BPH increased its droplet size during storage while suffering significant lipid oxidation. However, it was not able to prevent omega-3 oxidation in spite of in vitro radical scavenging or chelating effect compared to whey (WPH) or soy (SPH) protein hydrolysates	[147]
Silver carp	Surimi processing by-products (SPB; head, skin, fin, scale, bone, white muscle leftover on bones, and dark muscle)	SPB was heated at 121 °C for 2 h, and lyophilized powder was hydrolyzed with Alcalase (55 °C, pH 8) and trypsin (37 °C, pH 8) for 4 h	Peptides with MW < 0.5 kDa accounted for 40.4 and 47.9% in trypsin (DH 13.4%) and Alcalase (DH 18.0%), hydrolyzed after 4 h, respectively	Surimi	−18 °C/3 month	Partial replacement of sucrose with 2% trypsin and Alcalase hydrolysates effectively delayed the oxidation of Cys, the carbonylation of amino acids, the loss of Ca^2+^-ATPase activity, and the destruction of the structural integrity of myofibrillar protein	[148]
Silver carp	Fins	Fins were dried, dispersed in distilled water (1:5, *w*/*v*), and heated at 121 °C for 3 h, and the obtained gelatin was hydrolyzed using four enzymes (Alcalase: pH 8.0 and 50 °C; trypsin: pH 8.0 and 37 °C; neutrase: pH 7.0 and 45 °C; and papain: pH 7.0 and 55 °C) at 2% for 4 h	A total of 102 and 61 peptides below 2 kDa were identified in trypsin and Alcalase hydrolysates, respectively, and some of the identified peptides shared similar repeated structures to Gly-Pro-X, such as Gly-Asp-Thr-Gly-Hyp-Ser-Gly-Hyp-Leu, Hyp-Gly-Hyp-Ile-Gly-Hyp-Hyp-Gly-Hyp-Arg, and Gly-Gly-Arg-Gly-Hyp-Hyp-Gly-Glu-Arg	Bighead carp fillets	Fillets were immersed in 2% of Alcalase or trypsin hydrolysates with higher antioxidant activity and were frozen at −18 °C for 1 week and thawed at 4 °C (once a week, as one freeze–thaw cycle) and a total of six cycles	Protein oxidation (carbonyls and disulfide bonds) and degradation (the loss of Ca^2+^-ATPase activity), and lipid oxidation (PV, TBARS, FFA, and fluorescent compounds) were significantly inhibited by fin hydrolysates	[149]
Silver carp	Surimi processing by-products	By-products powders were hydrolyzed with Alcalase at 1:60 *w*/*w* at pH 8.5 and 55 °C	--	Surimi	Surimi mixed with 0.6 or 1.2% protein hydrolysates stored at −20 °C for 60 days	Surimi with protein hydrolysates showed lower TBARS, carbonyl content, and surface hydrophobicity; higher Ca^2+^-ATPase activity, total sulfhydryl groups, and salt-soluble proteins; and the reduced degradation of MP, thus inducing cross-linking more effectively, leading to the formation of a denser gel network	[150]
Silver carp	Muscle mince	Mince was hydrolyzed with Protamex (2400 U/g, pH 6.5, 50 °C) for 30 min and then fractionated into <3, 3–10, and >10 kDa fractions	DH was 13.6% and >50% peptides had an MW of 1000–2500 Da	Interactions between peptides and ice planes	Computational simulations	Gly-Val-Asp-Asn-Pro-Gly-His-Pro-Phe-Ile-Met, Gly-Val-Asp-Asn-Pro-Gly-His-Pro-Phe-Ile-Met-Thr, and Ile-Ile-Thr-Asn-trp-Asp-Asp-Met-Glu-Lys in the fractions with MW < 3 kDa interacted firmly with water molecules and inhibited the growth of ice crystals	[151]
Bighead carp (*Aristichthys nobilis*)	Gills	Gills were autoclaved (121 °C for 4 h to solubilize collagen) and hydrolyzed with Flavourzyme (pH 7.0), Alcalase (pH 8.0), neutral protease (pH 7.0), and papain (pH 7.0) at 5000 U/g for 4 h	Peptides with an MW of <0.5, 0–1, and 1–2 kDa were the dominant peptides, especially with increasing hydrolysis time	Surimi	Surimi (81% moisture) mixed with 1 or 2% neutral protease hydrolysates stored at −18 °C for 4 months	Surimi with hydrolysates had higher sulfhydryl and salt-soluble proteins and Ca^2+^-ATPase activity, lower disulfide bonds, carbonyls, and hydrophobicity	[152]
Common carp	Skin	Single-layer biopolymer films: furcellaran + carp skin gelatin hydrolysate; two-layer films: furcellaran + carp skin gelatin hydrolysate + Ala-Tyr	Synthetic Ala-Tyr peptide	Atlantic mackerel carcasses	Storage 4 °C, 15 days	Single- and double-layer coatings decreased lipid oxidation, but the addition of the peptide layer to the hydrolysate-furcellaran film did not improve its antioxidant effect	[153]
Pacific hake (*Merluccius productus*)	Fillets	Protamex (1%) was used to hydrolyze fillets for 1 h and with no pH adjustment (optimal condition)	Peptides were in the range of 95 to ~900 Da	Fish balls	Six freeze–thaw cycles (18 h at −25 °C and 6 h at 4 °C)	Protein hydrolysates decreased expressible moisture and cooking loss while maintaining salt-extractable proteins and the thermal properties of myosin	[154]

## Data Availability

The data that support this study are available from the corresponding authors upon request.
